# Correction: Sodium butyrate modulates chicken macrophage proteins essential for *Salmonella* Enteritidis invasion

**DOI:** 10.1371/journal.pone.0303452

**Published:** 2024-05-03

**Authors:** The PLOS One Staff

There are errors in Tables 1 and 5. In this study, the authors have shown differentially expressed proteins. However, due to errors in Table 1, the authors were not able to show upregulated proteins after *S*. Enteritidis infection in HTC cells. Similarly, in Table 5, due to deleted headings of upregulated proteins after sodium butyrate treatment in *S*. Enteritidis infection in HTC cells, the study could not be justified. Please see the correct Tables 1 and 5 here.

**Table 1:** Differentially regulated proteins in HTC cells after *S*. Enteritidis infection.

**Proteins** **(Downregulated proteins)**	**Alternate ID** **by Gene**	**UNIPROT** **Accession number**	**Molecular Weight**	**Fold change by category** **(SE/Control)**	**t-TEST** **(P-VALUE)** ***P*<0.05**
Uncharacterized protein	DNAH9	F1NVK1	482	0.2	0.032
Ryanodine receptor 2		F1NLZ9	563	0.2	0.035
Uncharacterized protein	NAV3	F1NAH8	250	0	0.0082
Biorientation of chromosomes in cell division 1 like 1	BOD1L1	R4GKR8	329	0	0.032
Actin-related protein 3	ACTR3	ARP3	47	0.4	0.0074
Zinc finger homeobox protein 4	ZFHX4	ZFHX4	395	0	0.0049
Non-specific serine/threonine protein kinase	ATM	E1C0Q6	348	0.2	0.022
Spectrin beta chain	SPTBN1	A0A1D5PJY1	274	0	0.013
Collagen type V alpha 2 chain	COL5A2	A0A1D5P6W1	145	0.1	0.029
Elongation factor 1-alpha	EEF1A1	A0A1L1RRR1	49	0.2	0.02
Zinc finger protein 462		E1C5J4	278	0	0.014
Actin-related protein 2/3 complex subunit 4	ARPC4	F1P010	20	0.3	0.049
Anaphase promoting complex subunit 1	ANAPC1	E1C2U7	216	0	0.024
Uncharacterized protein	CENPE	E1BQJ6	258	0	0.001
Uncharacterized protein	GMFB	A0A1D5PTE8	17	0.09	0.015
Uncharacterized protein	A0A1D5P0W7	A0A1D5P7P7	25	0.2	0.02
Natural killer cell triggering receptor	NKTR	A0A1D5PRM6	161	0	0.0069
Hsc70-interacting protein	ST13	A0A1L1RVN1	30	0	0.029
Terpene cyclase/mutase family member	LSS	A0A1D5PDR0	85	0	0.032
Adseverin OS = Gallus gallus	SCIN	A0A1D5PBC3	79	0.1	0.011
Uncharacterized protein	UBE2K	A0A1L1RJI2	22	0.1	0.0087
Histidine triad nucleotide binding protein 2	HINT2Z	R4GGS3	17	0	0.0076
**Proteins** **(Upregulated proteins)**	**Alternate ID** **by Gene**	**UNIPROT** **Accession number**	**Molecular Weight**	**Fold change by category** **(SE/Control)**	**t-TEST** **(P-VALUE)** ***P*<0.05**
Heat shock cognate 71 kDa protein	HSPA8	F1NWP3	71	1.2	0.0017
Bifunctional purine biosynthesis protein	ATIC	F7AXZ3	69	1.3	0.025
Peptidylprolyl isomerase	FKBP12	Q90ZG0	12	2	0.043
Hydroxymethylbilane synthase	HMBS	A0A1D5NYN8	37	2.6	0.04
EF-hand domain family member D2	EFHD2	A0A1D5PD25	25	3.5	0.017
Uncharacterized protein		A0A1D5P4K6	20	2.5	0.02
ATP synthase subunit d, mitochondrial	ATP5PD	E1C658	18	2	0.042
ATP synthase subunit alpha	ATP5A1	A0A182C637	60	1.3	0.039
Cytochrome c	CYC	CYC	12	12	0.03

**Table 5:** Differentially regulated proteins by sodium butyrate treatment in *S*. Enteritidis infected HTC cells.

**Proteins** **(Downregulated proteins)**	**Alternate ID** **by Gene**	**UNIPROT** **Accession number**	**Molecular Weight**	**Fold change by category** **(SB+SE/SE)**	**t-TEST** **(P-VALUE)** ***P*<0.05**
Alpha-actinin-1	ACTN1	A0A1D5P9P3	102	0.6	0.0084
Protein disulfide-isomerase	P4HB	PDIA1	57	0.6	0.038
Rab GDP dissociation inhibitor	GDI2	F1NCZ2	51	0.7	0.043
ATP-dependent 6-phosphofructokinase	PFKP	A0A1D5P0Z0	86	0.2	0.037
Vinculin	VCL	VINC	125	0.4	0.014
Uncharacterized protein	RCJMB04_4k19	Q5ZLW0	70	0.3	0.021
V-type proton ATPase catalytic subunit A	ATP6V1A	F1NBW2	68	0.2	0.017
Ubiquitin carboxyl-terminal hydrolase	UCHL3	F1NY51	22	0.2	0.03
Cathepsin D	CTSD	CATD	43	0.2	0.0092
NADPH—cytochrome P450 reductase	POR	F1P2T2	77	0.3	0.041
Uncharacterized protein	IDI1	F1NZX3	33	0.1	0.0041
WD repeat-containing protein 1	WDR1	F1NRI3	67	0.09	0.041
EF-hand domain family member D2	EFHD2	A0A1D5PD25	25	0.2	0.0066
Pyridoxal phosphate homeostasis protein OS	PROSC	E1C516	30	0	0.04
**Proteins** **(Upregulated proteins)**	**Alternate ID** **by Gene**	**UNIPROT** **Accession number**	**Molecular Weight**	**Fold change by category** **(SB+SE/SE)**	**t-TEST** **(P-VALUE)** ***P*<0.05**
Alpha-enolase	ENO1	A0A1L1RKH8	49	1.3	0.048
ATP synthase subunit beta, mitochondrial	ATP5F1B	ATPB	57	1.3	0.028
Ras-related protein Rab-11A	RAB11A	RB11A	24	2.2	0.03
Uncharacterized protein	HSPB9	A0A1L1RXQ8	21	2.4	0.013
Actin-related protein 2/3 complex subunit 4	ARPC4	F1P010	20	4.4	0.0023
Vimentin	VIM	VIME	53	5.6	0.03

The publisher apologizes for the errors.
